# *Mycobacteroides abscessus* complex vertebral osteomyelitis and discitis of the thoracolumbar spine: a systematic review of management and outcomes with illustrative case

**DOI:** 10.3389/fcimb.2026.1867912

**Published:** 2026-07-30

**Authors:** Andrew Schindler, Samuel B. Kankam, Audrey Demand, Tue Dinh, Yevgeniy Freyvert

**Affiliations:** 1 School of Engineering Medicine, Texas A&M University, Houston, TX, United States; 2 Department of Neurosurgery, Houston Methodist Neurological Institute, Houston, TX, United States; 3 Department of Plastic and Reconstructive Surgery, Houston Methodist Hospital, Houston, TX, United States

**Keywords:** discitis, immunocompetent, *Mycobacteroides abscessus* complex, non-tuberculous mycobacteria, rapidly growing mycobacteria, systematic review, vertebral osteomyelitis

## Abstract

**Introduction:**

*Mycobacteroides abscessus* complex (MAbC) is a rapidly growing non-tuberculous mycobacterium that can cause vertebral osteomyelitis in rare cases. This study systematically reviews the literature on MAbC vertebral osteomyelitis and presents a representative case.

**Methods:**

A Preferred Reporting Items for Systematic Reviews and Meta-Analyses (PRISMA)-guided review of PubMed/MEDLINE, Scopus, and Web of Science (from inception to December 2025) identified studies reporting MAbC vertebral osteomyelitis. Eligible cases included data on presentation, diagnosis, treatment, and outcomes. Risk of bias was assessed using Joanna Briggs Institute tools.

**Results:**

Twenty-three studies (28 patients) were included. The mean age was 56.2 years; 42.9% were male. Most patients presented with back pain, and 75.0% were immunocompetent. Antibiotic therapy was universal, and 70.0% underwent surgery. Younger age, female sex, granulomatous pathology, and surgical intervention were associated with improved outcomes (p < 0.05).

**Conclusion:**

MAbC vertebral osteomyelitis is rare and diagnostically challenging. Early tissue diagnosis and combined medical–surgical management are associated with improved outcomes, highlighting the importance of timely recognition and individualized treatment strategies.

**Systematic review registration:**

https://www.crd.york.ac.uk/PROSPERO/view/CRD420261333174, identifier CRD420261333174.

## 1 Introduction

*Mycobacteroides abscessus* complex (MAbC) vertebral osteomyelitis is a rare and diagnostically challenging form of spinal infection caused by a rapidly growing non-tuberculous mycobacterium (NTM), which is most commonly implicated in pulmonary disease, skin and soft-tissue infections, and postoperative or device-associated infections (Shimizu et al., 2013; Silva et al., 2015; Wessell et al., 2024). Epidemiological data on MAbC are generally derived from studies assessing all species of NTM, making it difficult to determine the specific epidemiological trends of *M. abscessus* (Cristancho-Rojas et al., 2024). However, the present literature demonstrates an overall increase in MABC infection and disease over the last decade (Cristancho-Rojas et al., 2024; Dahl et al., 2022). Spinal involvement typically presents with chronic or insidious back pain, and cross-sectional imaging may reveal vertebral body destruction, paravertebral or epidural abscess formation, or mixed lytic and sclerotic changes (Wessell et al., 2024; Yu et al., 2022). Neurological deficits can occur when inflammation or abscess extends to the epidural space, leading to neural compression (Farid et al., 2023; Kim et al., 2016; Omori et al., 2022; Shimizu et al., 2013; Silva et al., 2015; Wessell et al., 2024).

Although MAbC infections are more frequently reported in patients with underlying immunosuppressive conditions (including chronic corticosteroid use or advanced lung disease), vertebral osteomyelitis caused by this organism has also been documented in immunocompetent hosts (Kadota et al., 2019; Moral et al., 2019). The diagnosis is often delayed because the clinical and radiologic features are non-specific and may mimic *Mycobacterium tuberculosis*, pyogenic spondylodiscitis, or even metastatic malignancy (Wessell et al., 2024; Yu et al., 2022). Additionally, a recent study reported resistance to 76.7% of tested antibiotics, suggesting the presence of heteroresistance among the *M. abscessus* subspecies (Siew et al., 2024). Thus, definitive diagnosis requires microbiologic confirmation, typically through the isolation of the organism from image-guided biopsy or operative specimens. Polymerase chain reaction (PCR) can also be used as a diagnostic tool to distinguish organism subspecies and antibiotic resistance (Marras et al., 2021; Siew et al., 2024). Because *M. abscessus* frequently requires specialized media or extended incubation, multiple tissue samples may be necessary to achieve culture positivity. The current Infectious Diseases Society of America (IDSA) guidelines recommend obtaining a biopsy in cases of vertebral osteomyelitis when initial cultures are negative or when atypical pathogens are suspected (Berbari et al., 2015; Miller et al., 2024).

The management of MAbC vertebral osteomyelitis is complex due to the organism’s intrinsic and inducible antimicrobial resistance. Treatment usually involves prolonged, multidrug therapy guided by susceptibility testing and often includes a macrolide (when the *erm(41)* gene does not confer inducible resistance), combined with intravenous agents such as amikacin, imipenem, cefoxitin, or tigecycline (Silva et al., 2015). Oral agents such as linezolid or clofazimine may also be used depending on susceptibility and tolerance. Surgical intervention may be required for debridement, abscess drainage, and stabilization, particularly in cases complicated by neural compression or structural compromise (Bashti et al., 2023; Hirakawa et al., 2008; Kato et al., 2014; Omori et al., 2022; Patel et al., 2024; Rhodes et al., 2025). In addition, coordinated multidisciplinary care has been recommended for optimal treatment outcomes (Wessell et al., 2024). Despite these points, the factors that affect patient outcomes are poorly studied. Understanding the impact of various predictors on treatment outcomes could help tailor both medical and surgical interventions based on patient-specific factors. Thus, this systematic review aimed to compile existing literature to better understand the clinical presentation and management and to identify the predictors of patient outcomes for thoracolumbar vertebral osteomyelitis due to MAbC, complemented by an illustrative case.

## 2 Methods

We conducted a systematic literature search in accordance with the Preferred Reporting Items for Systematic Reviews and Meta-Analyses (PRISMA) 2020 guidelines (Page et al., 2021), which was registered with PROSPERO under number CRD420261333174 and available at https://www.crd.york.ac.uk/PROSPERO/view/CRD420261333174. An illustrative case is also provided, with appropriate consent obtained.

### 2.1 Search strategy

The authors queried PubMed/Medline, Scopus, and Web of Science for relevant studies from inception to December 3, 2025, without any language restrictions. The search strategy included the following keywords: (“Spine”[Mesh] OR spine OR spinal OR vertebra*) AND (“Mycobacterium abscessus”[Mesh] OR mabc OR “mycobacterium abscessus”) (**Supplementary Table 1**; Search strategy). The reference lists of relevant articles were also screened to identify additional studies.

### 2.2 Eligibility criteria

Studies were eligible for inclusion if they reported clinical cases or series of vertebral osteomyelitis and/or discitis caused by MAbC; involved infection of the thoracic, thoracolumbar, or lumbar spine; and provided sufficient clinical detail on diagnosis, management, and/or outcomes published in peer-reviewed journals. Studies were excluded if they involved non-spinal infections or extrapulmonary infections unrelated to the spine, focused on tuberculous or other non-tuberculous mycobacteria without specific identification of MAbC, or involved non-human subjects. Screening of the title, abstract, and full-text screening was independently performed by two researchers (A.S and S.B.K), with disagreement resolved through discussion or adjudication by a third reviewer (A.D).

### 2.3 Data extraction process

Data extraction was independently performed by two reviewers (A.S and S.B.K) using a standardized data collection form. Extracted variables included author information, study country, patient demographics (age and sex), comorbidities or prior risk factors, immune status (i.e., immunocompetent or immunocompromised), spinal level involved, clinical presentation, diagnostic modality or imaging, microbiologic confirmation, biopsy, antimicrobial regimen, the duration of therapy, surgical intervention, complications, and clinical outcomes. Discrepancies between reviewers were resolved through discussion and consensus. When necessary, a third reviewer (A.D) adjudicated unresolved disagreements.

### 2.4 Risk of bias

The risk of bias for the included studies was assessed using the Joanna Briggs Institute (JBI) Critical Appraisal Checklist for case reports and case series (Munn et al., 2020). Methodological quality was evaluated based on the clarity of patient history, diagnostic ascertainment, the completeness of intervention details, follow-up adequacy, and outcome reporting. Studies were categorized as follows per the JBI criteria: high quality (1 = “no” or 2 = “unclear” responses), moderate quality (2 = “no” or 2–4 = “unclear” responses), or low quality (≥2 = “no” or ≥4 = “unclear” responses). Any disagreements were resolved through reviewer consensus.

### 2.5 Statistical analysis

Given the rarity of MAbC vertebral infections and the predominance of case reports and small case series, quantitative meta-analysis was not feasible. Descriptive statistics were used to summarize the findings. Continuous variables were reported as means with standard deviations or medians with ranges, as appropriate. Categorical variables were summarized as frequencies and percentages. Based on the clinical course of patients reported by the individual studies, patient outcomes were categorized as improved, not improved, or not reported (NR). Then, cross-tabulation analyses were performed using Pearson’s chi-square test or Fisher’s exact test to identify the association between independent predictors and the dependent variable (clinical outcome). Additionally, for continuous variables, an independent test or ANOVA was conducted when needed. A p-value < 0.05 was considered statistically significant. Statistical analyses were conducted using STATA version 17.0 (StataCorp LLC).

## 3 Results

### 3.1 Study selection

**Figure 1:**
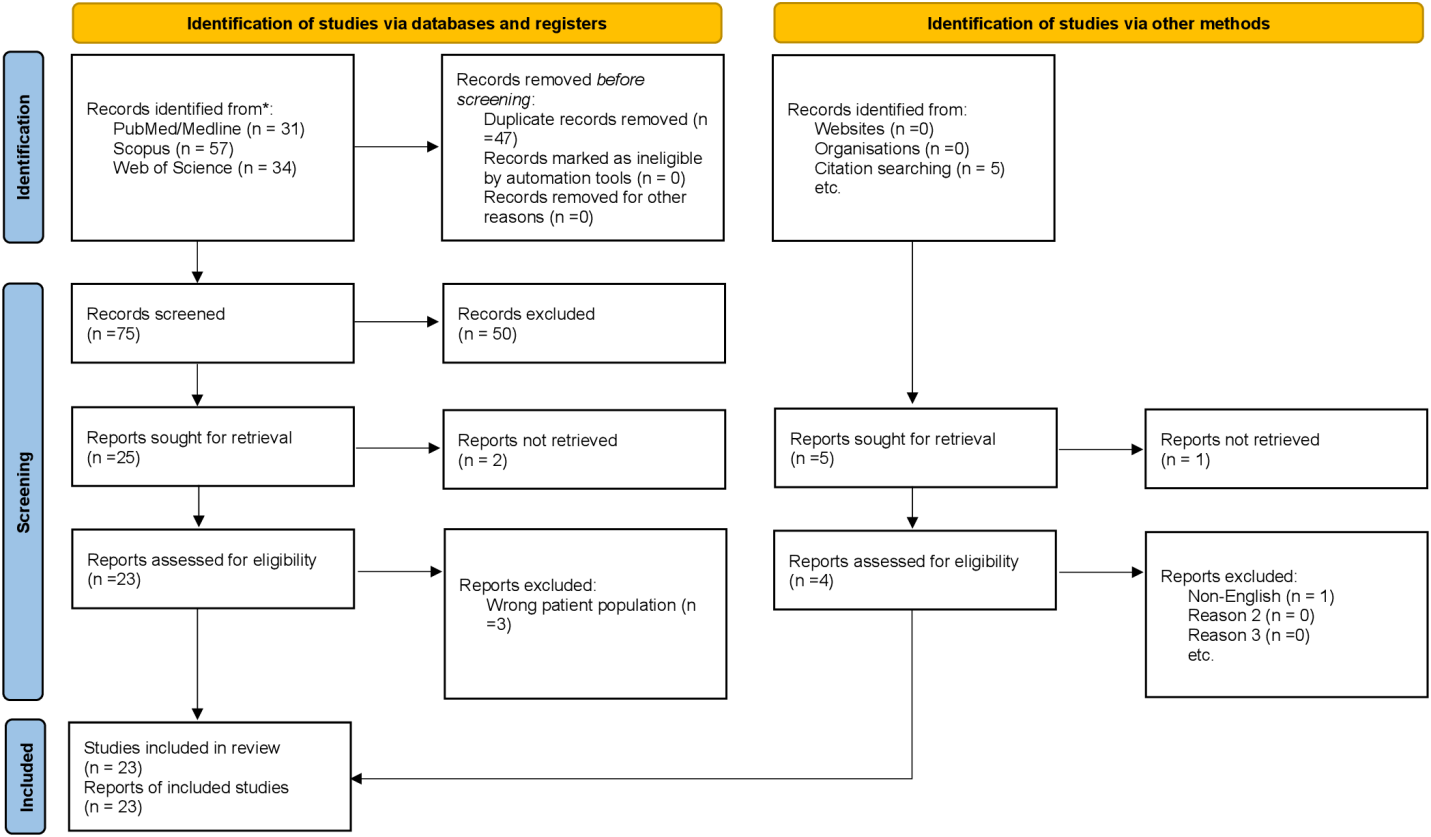
Preferred Reporting Items for Systematic Reviews and Meta-Analyses (PRISMA) flow diagram. This diagram shows the systematic process that we followed to include papers captured by our search. Alt text: Flow diagram illustrating the systematic review study selection process, including database identification, screening of titles and abstracts, full-text eligibility assessment, and final inclusion of studies analyzed.

A total of 122 records were identified from electronic databases. After the removal of 47 duplicates, 75 records remained for screening. Of these, 50 records were excluded based on title and abstract screening, and 25 reports were assessed for full-text review. Two articles could not be retrieved. Of the 23 full-text articles assessed for eligibility, three were excluded due to an inappropriate patient population, resulting in 20 studies identified from database sources (Bashti et al., 2023; Benjamin et al., 2024; Chan et al., 2001; Edwards et al., 2012; Farid et al., 2023; Garcia et al., 2013; Je et al., 2012; Kadota et al., 2019; Kato et al., 2014; Maxson et al., 1994; Moral et al., 2019; Omori et al., 2022; Patel et al., 2024; Rahi et al., 2021; Rhodes et al., 2025; Shahi et al., 2020; Sheikh et al., 2017; Silva et al., 2015; Singh et al., 2020; Zhang et al., 2018). Additionally, five articles were identified through citation screening; one could not be retrieved. Of the remaining four articles assessed for eligibility, one was excluded because it was a non-English draft, yielding three additional studies (Ma et al., 2021; Sarria et al., 1998; Serling-Boyd et al., 2020). In total, 23 studies met the inclusion criteria and were included in the qualitative synthesis (**Figure 1**).

### 3.2 Study characteristics and risk of bias assessment

A total of 23 studies comprising 28 patients diagnosed with and treated for MAbC vertebral osteomyelitis were identified. Publication years ranged from 1994 to 2025. Most of the included studies were case reports or small case series from diverse geographical regions, including the United States, Japan, Iran, the United Kingdom, China, Korea, India, Spain, and Australia. **Table 1** summarizes the demographic, clinical, radiographic, and management characteristics of patients with MAbC vertebral osteomyelitis. Quality appraisal using the JBI Critical Appraisal tools rated 16 studies as high quality, four as moderate quality, and three as low quality (**Supplementary Table 2**; study quality assessment).

**Table 1:** Summary of demographic, clinical, radiographical, and management characteristics of patients with MAbC vertebral osteomyelitis.

Author (Year)	Country	Age; Sex	Clinical Presentation	Immune Status	Initial Imaging Findings	Spinal Level	Prior Surgery / Risk Factors	Granuloma on Biopsy	Time to Diagnosis	Management	Outcome
Bashti et al. (2023)	USA	51; M	Falls (3d) and BLE (3m) radiculopathy, paresthesia, and numbness	Immunocompetent	MRI; enhancing collection at L2-3 ventral and to the left of the spinal canal causing severe compression of the thecal sac, heterogenous contrast enhancement of the L2-3 vertebral bodies and intervertebral disc	L2-L3	Chronic IV drug use, hepatitis C	none	NR	Surgical debridement + Antibiotic therapy	Complete symptomatic relief
Benjamin et al (2024)	UK	64; M	LBP, weight loss, night sweats	Immunocompetent	MRI	T8-9, L1-L2	history of CKD, IHD, HTN, cholecystectomy. Prior lumbar spine decompressive surgery	none	NR	NR	Symptoms resolution 1 year 2 months, last follow up Nov 2023 -> pt clinically stable
Chan et al. (2001)	USA	16; F	nonspecific resp symptoms + midthoracic back pain.	Immunocompetent	Radionuclide bone scan; increased uptake in T9 vertebral body. Spine MRI; soft tissue mass at T8-T10, enhancing T9 and T10	T8-T10	prior falls during competitive roller skating	necrotizing granuloma	~ 2 weeks	Antibiotic therapy	Symptom improvement 6 months
Edwards et al. (2012)	USA	50; F	LBP and left leg numbness	Immunocompetent	MRI; enhancing paravertebral soft tissue at L4-L5 + epidural abscess	L4-L5	prior lumbar laminectomy	NR	NR	Antibiotic therapy	Lost to follow up
Farajzadeh Sheikh et al. (2017)	Iran	NR	LBP	Immunocompetent	MRI	L3	Addict, with prior TB close contact	NR	NR	NR	NR
Farajzadeh Sheikh et al. (2017)	Iran	NR	LBP	Immunocompetent	MRI	L4	DM, renal failure	NR	NR	NR	NR
Farajzadeh Sheikh et al. (2017)	Iran	NR	LBP	Immunocompetent	MRI	L3	DM, Metastatic lung cancer	NR	NR	NR	NR
Farajzadeh Sheikh et al. (2017)	Iran	NR	LBP	Immunocompetent	MRI	L4-L5	DM	NR	NR	NR	NR
Farajzadeh Sheikh et al. (2017)	Iran	NR	Fever, LBP, paraspinal abscess	Immunocompetent	MRI	L3-L4	None	NR	NR	NR	NR
Farid et al., (2023)	Japan	38; M	LBP for 1 year; b/l thigh pain	Immunocompetent	CT scan; anterior-dominant bone destruction at L1-L2	L1-L2	IV drug use, tattooing	none	NR	Surgical debridement + Antibiotic therapy	Symptom resolution at 1 year F/U
Garcia et al., (2013)	USA	44; M	night sweats, LBP for 1 year, radicular pain into the legs	Immunocompromised	Radiographs; loss of disc height at L4-S1, partial collapse at the L5 vertebral body. MRI; discitis at L4–5 level with soft tissue enhancement	L4-L5	Hepatitis C (2 year history)	none	NR	Antibiotic therapy	Complete resolution at 1 year follow up
Je et al. 2012	Korea	55; F	LBP for 10 months	Immunocompetent	CT scan; infectious spondylitis of L2 and L3 and disc bulging with annular tears at L3-L4 and L5-S1	L3-L4	None	none	NR	Surgical debridement + Antibiotic therapy	Complete resolution at 2mo follow up
Kadota et al., (2019)	Japan	63; F	LBP persisting for 4 months and a 2-day history of fever and right chest pain	Immunocompetent	CT scan; a right-sided pleural effusion	T7-T8	None	NR	NR	Antibiotic therapy	No recurrence or symptoms at 2 years
Kato et al., (2014)	Japan	67; F	LBP and a low-grade fever of 3-month	Immunocompetent	CT scan; destructive changes of L1 and L2	L1-L2	hypothyroidism	none	NR	Surgical debridement + Antibiotic therapy	Successful fusion with no symptoms at 2 years
Maxson et al., 1994	USA	21; F	LBP for 1 year; pain in her groin and left thigh with radiation to her left knee.	Immunocompromised	Roentgenogram; worsening of the L2 vertebral body fracture with a new compression fracture at L1	L1-L2	SLE, chronic steroid use (prednisone), prior L2 compression fracture	none	POD 12, ~ 3mo after initial presentation	Surgical debridement + Antibiotic therapy	clinically cured at 7mo F/U. Developed high-frequency SHL bilaterally (amikacin). No evidence of recurrent infection
Moral et al., (2019)	USA	82; M	recurrence of LBP with progressive functional impairment	Immunocompetent	MRI; worsening L1 vertebral body collapse, a T12 inferior endplate fracture, and a L2 superior endplate fracture	T12-L1	Remote history of prior falls, recent traumatic compression fracture of L1	none	6 weeks after biopsy	Surgical debridement + Antibiotic therapy	Aggressive care withdrawn, palliative measures only at follow up
Omori et al., (2021)	Japan	74; M	mild LBP for five months; paralysis in the lower limbs bilaterally and bladder-rectal disorder on the day of admission	Immunocompromised	CT scan; an additional collapse of vertebral body T7	T5 and T7	Lung cancer and prednisolone treatment	none	NR	Antibiotic therapy	Expired due to deteriorating general condition
Patel et al., (2024)	USA	63; F	LBP radiating to the bilateral lower extremities	Immunocompetent	CT scan and MRI (abdomen/pelvis); L2–L3 discitis-osteomyelitis with a large anterior epidural abscess	L2-L3	Lumbar vertebral osteomyelitis, bilateral psoas abscesses, epidural abscess	caseating granuloma	NR	Surgical debridement + Antibiotic therapy	intact neurological function and functional status, including the ability to ambulate and perform activities of daily living at 1 month
Rahi et al., (2021)	USA	55; M	LBP exacerbation	Immunocompromise	CT scan (chest); bilateral cavitating nodules	L4-L5	Hepatitis C, liver cirrhosis, intravenous drug use	none	NR	Antibiotic therapy	Clinically doing well after 1 month follow up
Rhodes et al., (2025)	USA	76; M	LBP and left-sided radicular symptoms.	Immunocompetent	MRI; a compressive dorsolateral fluid collection at the L5–S1 interspace and left S1 pedicle	L5-S1	L2-5 LLIF, L5-S1 TLIF and extension of prior fusion from L2-S1	none	POD 60	Surgical debridement + Antibiotic therapy	Improved ambulation, no radicular symptoms, normalized inflammatory markers, thrombocytopenia and splenomegaly w/o associated LAP
Shahi et al., (2020)	India	43; F	Severe LBP, BLE radiculopathy, and a partial right-sided foot drop	Immunocompetent	MRI; L4–L5 spondylodiscitis with para-discal erosion	L4-L5	Fluoroscopically guided percutaneous ozone treatment for degenerative disc disease	none	NR	Surgical debridement + Antibiotic therapy	resolution of primary infection
Silva et al., (2015)	Spain	71;F	LBP, fever, neutropenia	Immunocompromised	MRI; osteoporotic vertebral compression fractures (T5–T7, T9–T12, L1, L3, and L4), abnormal T6 body signal, with perivertebral soft-tissue swelling	T6	Orthotopic heart transplantation and subsequent immunosuppressive therapy	none	NR	Antibiotic therapy	Asymptomatic without recurrence of vertebral disease at 11 months
Singh et al. 2020	USA	60; NR	acute worsening of chronic LBP	Immunocompetent	MRI; L4-5 diskitis and osteomyelitis with concern for epidural abscess	L4-L5	intradiskal ozone and stem cell injections into the L4-5 disk 2 mo prior in Mexico	none	NR	Antibiotic therapy	clinical improvement
Zhang et al., (2018)	AU	51; M	5 days of fever, cough, shortness of breath and pleuritic chest pain	Immunocompromised	CT scan; right lower lobe consolidation and parapneumonic effusion and osteolytic lesions of the T9/10 vertebral bodies immediately adjacent to the effusion	T8-T10	cell injections into the L4-5 disk	none	6 weeks after biopsy	Surgical debridement + Antibiotic therapy	No hardware complications, no progression of infection, no neurological deficits, ambulatory at 10 months post op
Serling-Boyd et al., (2020)	USA	80; M	fever, joint pain, and weakness	immunocompetent	Chest XR and CT; no focal findings. MRI; T2 hyperintensity in the L4–L5 disc space with associated end plate edema, suggestive of discitis and/or osteomyelitis	L4-L5	B/L shoulder arthroplasty, right hip arthroplasty, MGUS	none	NR	Antibiotic therapy	Hospice after 3 months of antibiotic treatment
Ma et al. (2021)	China	75; F	Lumbar disc protrusion with lumbar vertebral body destruction with abscess	NR	MRI; signal abnormality in second and third lumbar vertebral bodies with abscess formation	NR	None	granuloma	7-7.5 month	Surgical debridement + Antibiotic therapy	Cured
Sarria et al. (1998)	USA	53; M	Low-back pain, malaise, night sweats, and a 15-lb weight loss	Immunocompetent	MRI; extensive bone destruction and collapse of the L3, L4, and L5 vertebral bodies + paravertebral abscess	L3-L5	Prior IV cocaine use	NR	**NR**	Surgical debridement + Antibiotic therapy	Clinical improved but walk with aid
**Present case (2025)**	**USA**	**41; M**	**poor appetite, malaise,** LBP**, and chills**	**Immunocompetent**	**MRI; L4-L5 osteomyelitis and discitis**	**L4–L5**	**Prior microdiscectomy (postoperative seroma)**	**granuloma**	**1 month**	Surgical debridement + Antibiotic therapy	**Ongoing improvement**

M, male; F, female; LBP, low back pain; BLE, bilateral lower extremity; MRI, magnetic resonance imaging; XR, x-ray; CT, computed-tomography; NR, not reported; LAP, lymphadenopathy; POD, post-operative day

### 3.3 Patient demographic and infection characteristics

Of the 28 patients, 12 (42.9%) were male, and 10 (35.7%) were female; sex was unreported in six patients (21.4%). The mean age was 56.2 years (range, 16–82 years). The majority of patients (n = 16, 57.1%) presented with low back pain accompanied by constitutional or radicular symptoms, while 12 patients (42.9%) presented with isolated low back pain. Most patients were immunocompetent (75.0%). All included studies reported MAbC vertebral osteomyelitis involving the thoracolumbar region, with no cases affecting the cervical or sacral spine. Major predisposing risk factors were present in 24 patients (85.7%), whereas four patients (14.3%) had no documented risk factors. Biopsy was performed in 20 patients (71.4%), and biopsy status was unreported in eight patients (28.6%). Among the 20 biopsies performed, granulomatous pathology was identified in six cases (30.0%), while 14 cases (70.0%) showed no granuloma (**Table 2**).

**Table 2:** Demographic, clinical and treatment outcomes.

	Improved Outcome at Last F/U			
Variables*	No	Yes	NR	p-Values^§^
Age	78.67±4.16	51.71±16.44	50	0.040
Sex (%)				<0.001
Male	3 (25.0)	9 (75.0)	0	
Female	0	9 (90.0)	1 (10.0)	
Missing data	0	1 (16.7)	5 (83.3)	
Clinical presentation (%)				0.503
Low back pain only	1 (8.4)	7 (58.3)	4 (33.3)	
Low back pain with other symptoms^‡^	2 (12.5)	12 (75.0)	2 (12.5)	
Immune status (%)				0.566
Immunocompromised	1 (16.7)	5 (83.3)	0	
Immunocompetent	2 (9.5)	13 (61.9)	6 (28.6)	
Missing data	0	1 (100.0)	0	
Spine Location (%)				0.200
Thoracic only	1 (20.0)	4 (80.0)	0	
Lumbar only	1 (4.7)	14 (66.7)	6 (28.6)	
Thoracolumbar	1 (50.0)	1 (50.0)	0	
Risk factors (%)				1.00
No	0	3 (75.0)	1 (25.0)	
Yes	3 (12.5)	16 (66.7)	5 (20.8)	
Biopsy findings (%)				<0.001
Biopsy without granuloma	3 (21.4)	11 (78.6)	0	
Biopsy with granuloma	0	6 (100.0)	0	
Missing data	0	2 (25.0)	6 (75.0)	
Surgical debridement (%)				<0.001
No	1 (14.3)	6 (85.7)	0	
Yes	2 (14.3)	12 (85.7)	0	
Missing data	0	1 (14.3)	6 (85.7)	

*Data is presented in count, frequency with percentage for categorical variables and mean ± SD for continuous variables.‡NR: not reported, §p values <0.05 are considered statistically significant.

### 3.4 Treatment and outcomes

**Figure 2:**
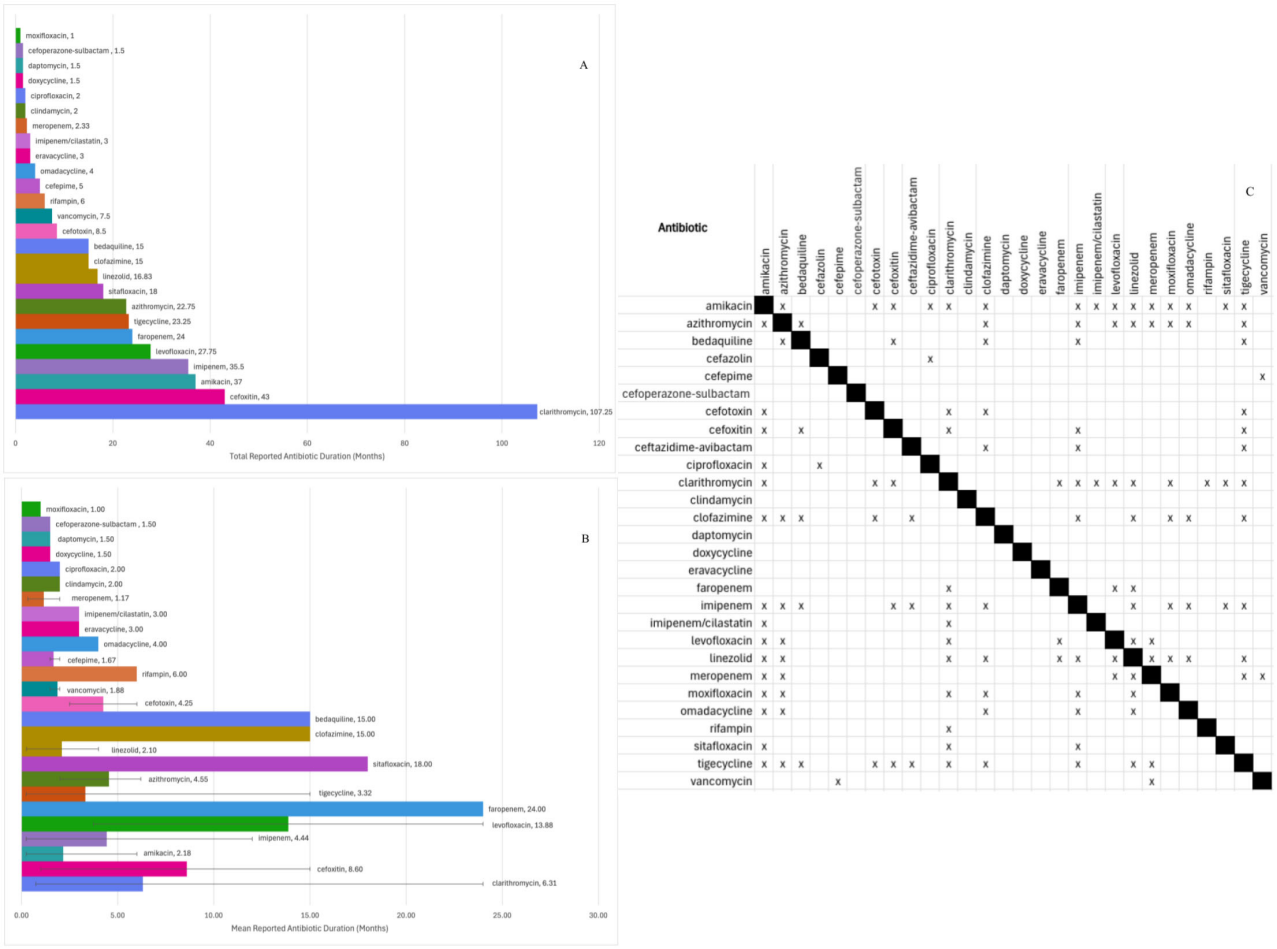
Duration of antibiotic use in months. All antibiotics used for the 28 patients in this literature review were recorded along with treatment durations and drug combinations. **(A)** The total reported times of each antibiotic in months across the 28 different patients in this review. Of note, ethambutol, piperacillin/tazobactam, ceftazidime/avibactam, cefazolin, isoniazid, and ceftriaxone were also used, with no reported durations. Two antifungals (anidulafungin and fluconazole) were also reported. This does not account for differences in combination therapies, treatment sequences, or duration of administration across studies and is purely an illustration to display the most commonly used antibiotic therapies. **(B)** The mean reported antibiotic treatment time per regimen. The mean duration was calculated by dividing the total time of treatment for each antibiotic by the number of instances each was included in and reported in an antibiotic treatment regimen. Range bars depict the minimum and maximum reported durations for each antibiotic. **(C)** A reported antibiotic combination matrix demonstrating concurrent antimicrobial regimens used in reported cases. Each marked cell represents a documented combination of the corresponding antibiotics within an individual treatment regimen. Alt text: Three-panel figure summarizing antibiotic use in 28 patients with *Mycobacteroides abscessus* complex (MAbC) vertebral osteomyelitis: **(A)** total cumulative duration (months) of each antibiotic across cases, **(B)** mean duration per antibiotic regimen, with some agents included without reported durations, and **(C)** an antibiotic combination matrix showing reported antibiotic combinations.

Antibiotic therapy was a key component of management and was reported in most studies (**Figure 2**). In addition, 14 patients (70.0%) underwent surgical intervention, whereas seven patients (25.0%) received antibiotic therapy alone; treatment details were not reported for seven patients (25.0%). In the cross-tabulation analysis, younger patients demonstrated significantly greater clinical improvement compared with older patients (mean age 51.7 vs. 78.7 years; p = 0.040), and female sex was also significantly associated with improved outcomes (p < 0.001). The presence of granulomatous pathology on biopsy showed a strong association with clinical improvement (p < 0.001), and surgical debridement was similarly strongly associated with improved outcomes (p < 0.001), whereas non-operative management was associated with poorer outcomes. In contrast, clinical presentation (p = 0.503), immune status (p = 0.566), the spinal location of a disease (p = 0.200), and the presence of baseline risk factors (p = 1.000) were not significantly associated with clinical outcome (**Table 2**).

### 3.5 Case presentation

A 41-year-old immunocompetent man with a history of lumbar radiculopathy and prior L4–L5 microdiscectomy complicated by postoperative infection presented to our institution for higher-level care with several months of purulent wound drainage, chills, malaise, and minimal back pain. He had no history of immunodeficiency, chronic steroid use, or pulmonary disease. His index surgery was performed 15 months prior at an outside hospital, followed by postoperative seroma formation with wound dehiscence and systemic symptoms. Four months later, his MRI showed L4–L5 osteomyelitis/discitis with a small paraspinal abscess. CT-guided aspiration yielded only skin flora (*Corynebacterium*), and he completed an 8-week course of intravenous vancomycin.

Despite therapy, his symptoms worsened, with interval progression of infection observed on MRI. He underwent surgical washout 9 months prior to presentation at our institution with negative intraoperative cultures, followed by recurrent wound dehiscence and purulent drainage. He completed an additional 8-week course of intravenous vancomycin and cefepime with oral clindamycin under infectious disease guidance. He improved transiently until 1 month prior to presentation, when recurrent symptoms prompted a repeat MRI showing progressive L4–L5 endplate destruction and a small abscess extending to L3. He then presented to our institution for higher-level care (Day 1).

**Figure 3:**
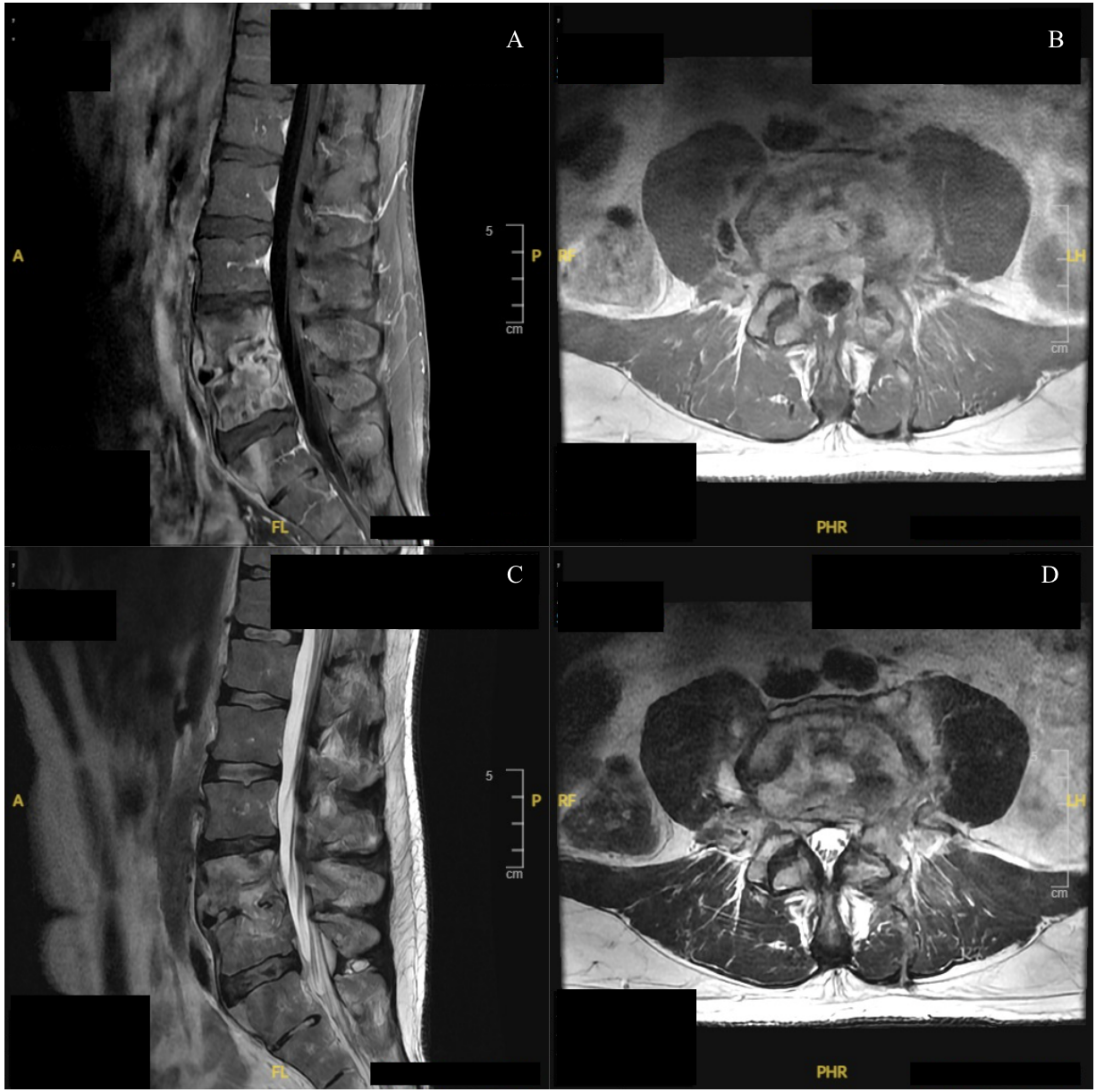
Initial presentation MRI showing chronic L4–L5 spondylodiscitis and abscess. MRI on presentation demonstrating chronic L4–L5 spondylodiscitis with enhancing endplate necrosis and drainage into the disc space. Infection extends to the L5–S1 disc with anterior S1 osteomyelitis and suspected involvement of the L3–L4 disc space. Bilateral psoas and left paraspinal abscesses are present, with a 4 mm L4–L5 abscess/phlegmon and extension of inflammation into the bilateral L4–L5 and L5–S1 neural foramina. **(A)** Sagittal T1 post-contrast MRI, **(B)** axial T1 post-contrast MRI, **(C)** sagittal T2 image, and **(D)** axial T2 image. Alt text: MRI of the lumbar spine demonstrating chronic L4–L5 spondylodiscitis with endplate destruction, disc space involvement, and extension to L5–S1 and possibly L3–L4, along with bilateral psoas and left paraspinal abscesses and inflammatory involvement of adjacent neural foramina (sagittal and axial T1 post-contrast and T2 images).

Throughout his course, laboratory studies showed normal to mildly elevated white blood cell counts and modestly elevated inflammatory markers [white blood cell (WBC), 7, 940/µL; erythrocyte sedimentation rate (ESR), 22 mm/hr; C-reactive protein (CRP), 1.39 mg/dL]. Neurological examination was normal, with full strength and no radicular or myelopathic symptoms. MRI confirmed chronic L4–L5 spondylodiscitis with associated abscess (**Figure 3**).

### 3.6 Therapeutic intervention

The patient underwent wound exploration and debridement with tissue sampling and tissue culture 6 days later. Following the wound exploration, cultures were positive only for *Staphylococcus pseudintermedius*. A peripherally inserted central catheter (PICC) line was placed, and he was discharged 2 days later with a 6-week course of daptomycin and cefepime. Notably, results for fungal, acid-fast bacillus (AFB), and PCR analyses were pending at the time of discharge. The PCR analysis did not detect any bacterial, fungal, or mycobacterial organisms.

**Figure 4:**
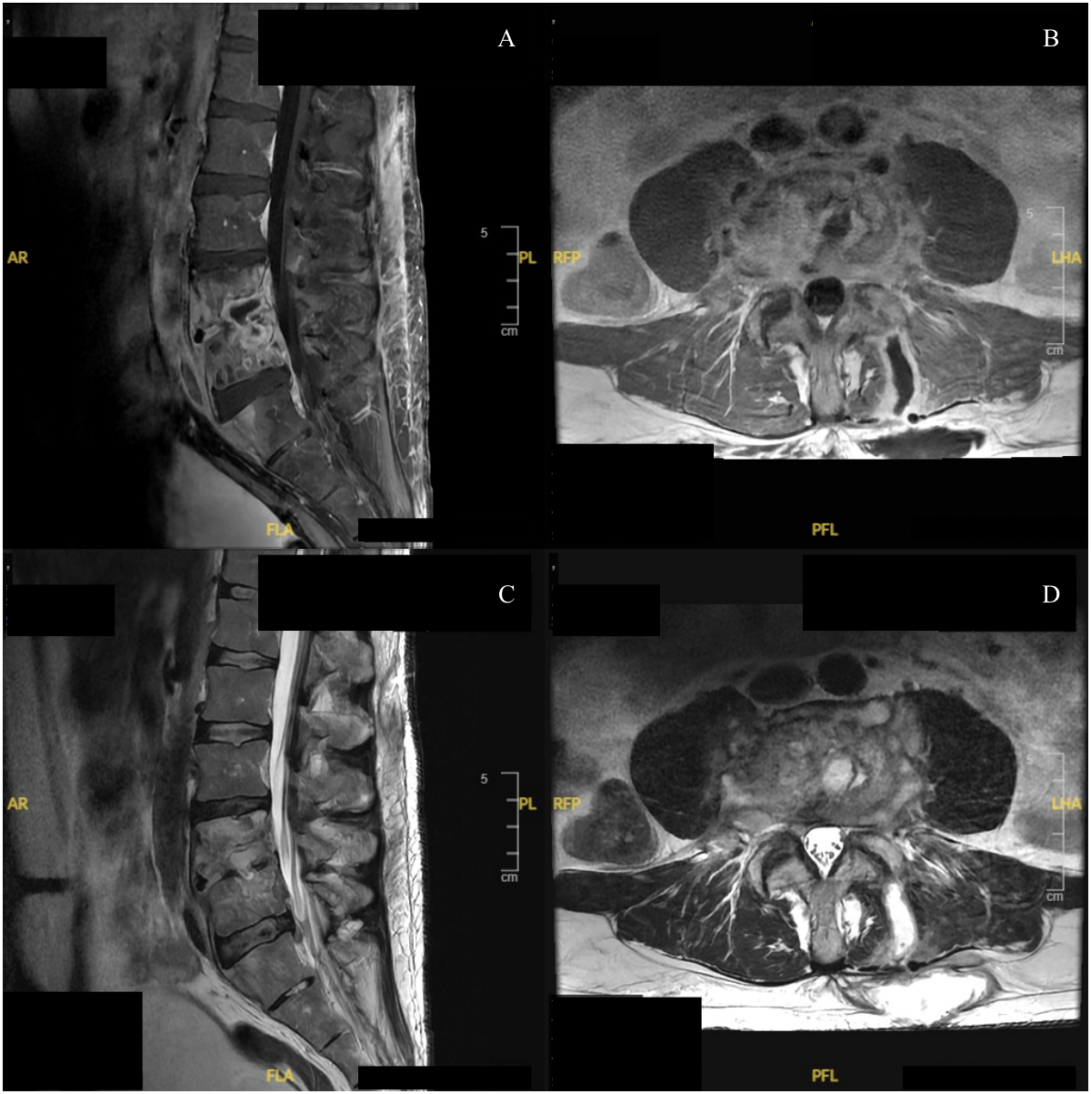
Day 29 MRI showing interval worsening. Repeat MRI on Day 29 after re-presentation with purulent drainage demonstrated persistent L4–L5 infection with progression of paraspinal, epidural, and foraminal abscesses. The left posterior L4–L5 fluid collection increased to 2.8 × 0.8 × 3.8 cm, with a new subcutaneous fluid collection extending from L1–L2 to L5–S1 measuring 3.8 × 1.6 × 13 cm. **(A)** Sagittal T1 post-contrast MRI, **(B)** axial T1 post-contrast MRI, **(C)** sagittal T2 image, and **(D)** axial T2 image. Alt text: Follow-up lumbar spine MRI (Day 29) showing progression of infection with persistent L4–L5 spondylodiscitis, enlarging paraspinal abscess, and new extensive subcutaneous fluid collection extending from L1–L2 to L5–S1, with involvement of epidural and foraminal regions (sagittal and axial T1 post-contrast and T2 images).

He returned 3 weeks after his initial discharge with recurrent purulent drainage and worsening infection observed on MRI (**Figure 4**). On Day 26, AFB culture results indicated *M. abscessus*, which was identified using matrix-assisted laser desorption/ionization time-of-flight mass spectrometry (MALDI–TOF MS), and antimicrobial therapy was transitioned to a four-drug regimen consisting of amikacin, azithromycin, imipenem, and eravacycline with close laboratory and ECG monitoring. Additional washout was performed 6 days later. Susceptibility testing confirmed macrolide sensitivity due to a non-functional *erm(41)* gene, and all agents except imipenem were maintained.

Recurrent purulent drainage necessitated additional washout on Days 68 and 93, with placement and subsequent replacement of vancomycin/amikacin antibiotic spacers. The PICC line was removed on Day 114, and therapy was transitioned to oral agents. The final AFB cultures were negative. His final surgery 1 week later included the removal of antibiotic beads, repeat cultures, and closure using a local longissimus muscle flap to obliterate paraspinal dead space.

### 3.7 Follow-up and outcomes

**Figure 5:**
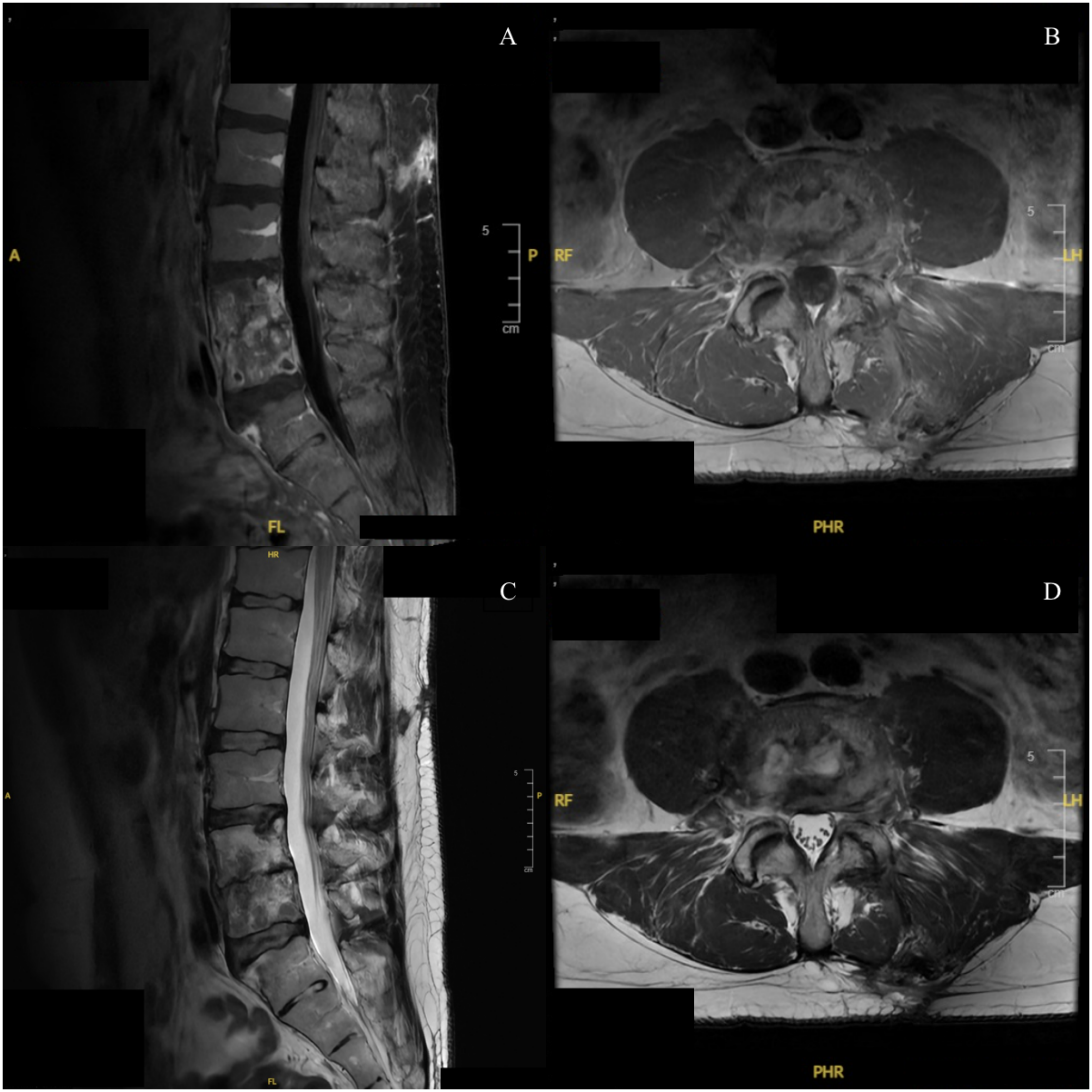
Latest follow-up MRI showing considerable improvement. Repeat MRI at the latest follow-up (Day 235) demonstrated marked improvement in the previously identified paraspinal and bilateral psoas abscesses, with decreased edema and enhancement involving the L4–L5 vertebral bodies, L4–L5 disc, and anterior S1 vertebral body. Epidural enhancement posterior to L4 and L5 also improved significantly, although residual enhancement persisted in the left greater than right L4–L5 neural foramina. **(A)** Sagittal T1 post-contrast MRI, **(B)** axial T1 post-contrast MRI, **(C)** sagittal T2 image, and **(D)** axial T2 image. Alt text: Follow-up lumbar spine MRI (Day 235) demonstrating marked improvement of prior L4–L5 spondylodiscitis, with resolution of paraspinal and psoas abscesses, decreased vertebral and disc edema, and reduced epidural enhancement, with minimal residual enhancement in the L4–L5 neural foramina.

At the last follow-up, MRI showed marked radiographic improvement (**Figure 5**), with planned repeat imaging scheduled in 9 months. The patient reported mild back stiffness without neurological symptoms or wound complications. He experienced dermatologic changes, transiently impaired appetite, and mild peripheral neuropathy attributed to antimycobacterial therapy. He has returned to work full-time, resumed physical therapy, and continued routine follow-up with neurosurgery and infectious disease services (**Supplementary Table 3**; Timeline of events).

## 4 Discussion

Vertebral osteomyelitis caused by MAbC is exceptionally rare, particularly among immunocompetent individuals. Most reported cases occur in patients with underlying pulmonary disease, chronic corticosteroid exposure, or prior spinal procedures (Shimizu et al., 2013; Silva et al., 2015; Wessell et al., 2024). The pathogenesis in immunocompetent hosts remains incompletely understood but may involve direct inoculation during spinal surgery, postoperative wound complications, or hematogenous seeding following transient bacteremia. In this case, the patient’s history of lumbar microdiscectomy and postoperative seroma likely created a permissive local environment for bacterial introduction and subsequent infection despite otherwise unimpaired systemic immunity. The findings of our systematic review support this observation, as prior spinal surgery or localized tissue disruption was a common feature across multiple reported cases, even in the absence of systemic immunosuppression.

Diagnosing MAbC vertebral osteomyelitis presents substantial challenges. Symptoms are often insidious and non-specific, with worsening back pain being the most common presenting feature, although this was not noted in the present case. The absence of an elevated WBC count should not rule out a differential diagnosis of spinal infection. In spinal epidural abscess, approximately 60% of patients demonstrate leukocytosis, and although our patient had a paravertebral rather than epidural abscess, a similar principle likely applies (Rice-Canetto et al., 2025). Consistent with reports in the literature, our patient exhibited only mild inflammatory marker elevations, with ESR and CRP proving more sensitive than WBC count for detecting ongoing infection (Gutovitz et al., 2025; Sheikh et al., 2017).

Routine bacterial cultures frequently yield no growth, contributing to diagnostic delays. MRI findings, including progressive vertebral body destruction, disc space involvement, and paravertebral or epidural abscess formation, are not unique to *M. abscessus* and overlap significantly with those of *M. tuberculosis* and pyogenic spondylodiscitis (Yu et al., 2022). Therefore, additional diagnostic consideration, including microbiologic testing, is warranted. In the present case, microbiologic confirmation was achieved only after surgical debridement and multiple intraoperative tissue specimens, highlighting the importance of aggressive pursuit of tissue diagnosis in culture-negative or atypical spinal infections. Several reports identified in our systematic review similarly required repeat biopsies or prolonged incubation before organism identification (Bashti et al., 2023; Benjamin et al., 2024; Edwards et al., 2012; Farid et al., 2023; Garcia et al., 2013; Kadota et al., 2019; Kato et al., 2014; Moral et al., 2019; Omori et al., 2022; Rhodes et al., 2025). In our review, granulomatous pathology on biopsy was associated with improved patient outcomes. The presence of granulomatous inflammation on biopsy may reflect a more effective, organized host immune response to mycobacterial infection, potentially aiding in infection containment. Withholding antibiotics prior to biopsy, when clinically feasible, may improve diagnostic yield and should be considered in suspected cases of atypical infection. Rapidly growing non-tuberculous mycobacteria should remain part of the differential diagnosis when conventional diagnostics fail.

The management of MAbC vertebral osteomyelitis is further complicated by the organism’s intrinsic multidrug resistance and its characteristic inducible macrolide resistance mediated by the *erm(41)* gene (Silva et al., 2015). Consequently, treatment must be individualized and guided by susceptibility testing, consistent with IDSA recommendations for extrapulmonary rapidly growing non-tuberculous mycobacterial disease (Berbari et al., 2015; Miller et al., 2024). Standard regimens used for other NTMs—such as macrolide, ethambutol, and rifampin therapy for *Mycobacterium avium* complex (MAC)—are inappropriate for MAbC. Instead, therapy typically involves prolonged combination treatment with a macrolide (if susceptibility is confirmed), intravenous amikacin, and additional agents such as imipenem, cefoxitin, tigecycline, clofazimine, or linezolid, tailored to resistance patterns, patient tolerance, and route of administration (Kadota et al., 2019; Kato et al., 2014). Emerging therapies for refractory NTM infections include the use of new agents such as bedaquiline, omadacycline, eravacycline, and clofazimine-based salvage regimens, although clinical experience in spinal infections remains limited (Bi et al., 2015). In our review, the most commonly reported antibiotics were clarithromycin and amikacin (each reported 17 times). Imipenem, linezolid, tigecycline, cefoxitin, and azithromycin were also commonly reported (8, 8, 7, 5, and 5 times, respectively). The findings of our systematic review reveal significant heterogeneity in antimicrobial regimens and duration, reflecting the absence of spine-specific guidelines and the need for individualized, multidisciplinary management (**Figure 2**).

Surgical intervention was associated with improved outcomes in our review and plays a central role in the management of MAbC vertebral osteomyelitis, particularly given the organism’s propensity for biofilm formation and persistence within necrotic tissue. Progressive neurological deficits (often due to spinal cord or nerve root compression), progressive deformity, abscess drainage, and spinal instability should be considered when determining whether surgical intervention is appropriate (Miller et al., 2024). Albeit a weaker recommendation for surgery, worsening pain despite appropriate antimicrobials can also be a consideration (Miller et al., 2024). Importantly, radiographic progression can occur even with successful medical treatment; therefore, imaging changes alone should not drive surgical decisions if other parameters (clinical symptoms, physical exam, and inflammatory markers) indicate clinical improvement (Miller et al., 2024). Across the reviewed literature, most patients required surgical debridement for diagnostic confirmation, infection control, or spinal stabilization (70%) (Miller et al., 2024). Surgical debridement likely contributes to improved outcomes by reducing bacterial burden, disrupting biofilm, and improving antibiotic penetration. In the present case, serial washouts combined with antibiotic spacer placement and eventual muscle flap closure were necessary to achieve disease control. Notably, we were able to avoid significant bony resection, which would have necessitated hardware placement with associated risk of biofilm formation and consequent long-term antibiotic treatment. These findings align with published reports demonstrating improved outcomes when surgical debridement is combined with prolonged, susceptibility-guided antimicrobial therapy (Kato et al., 2014).

Two additional factors identified in our systematic review, patient gender and age, were significantly associated with improved clinical outcomes in MAbC vertebral osteomyelitis and warrant further consideration. Although evidence specific to rapidly growing NTM remains limited, sex-based immunologic differences have been described in mycobacterial infections, typically describing male individuals as disproportionally affected, with higher mortality rates (Gebrie et al., 2026). Our study underscores this research, demonstrating that female gender is associated with improved outcomes (Chidambaram et al., 2021; Gebrie et al., 2026; Rickman et al., 2025). Additionally, we found an association between younger age and improved outcomes. Younger patients likely benefit from greater physiologic reserve and immune responsiveness, leading to significantly lower mortality rates and fewer treatment failures in patients with vertebral osteomyelitis (Courjon et al., 2017). Together, our findings support that several host factors (including gender, age, and granulomatous pathology), combined with medical–surgical management, lead to optimized outcomes in patients with MAbC thoracolumbar vertebral osteomyelitis.

Advanced and adjunctive therapies for NTM infections are increasingly being explored. Investigational approaches include nanoparticle-based drug delivery systems, efflux pump inhibitors, antimicrobial peptides, bacteriophage therapy, and phytochemicals, all aimed at improving antimicrobial efficacy. Other emerging modalities include inhaled nitric oxide, small molecule therapeutics, phototherapy, and immunomodulatory therapy. Although most remain experimental and not specific to MAbC vertebral osteomyelitis, these strategies highlight the growing range of potential treatments for refractory NTM infections (Sreekumar et al., 2024).

This review has several important limitations. First, vertebral osteomyelitis due to MAbC is very rare, and the available literature is largely limited to isolated case reports and small case series. Consequently, a quantitative meta-analysis was not appropriate, and descriptive statistics were used to summarize the data available. Second, due to the small sample size, substantial heterogeneity in patient characteristics, diagnostic approaches, antimicrobial regimens, surgical strategies, outcome reporting, and follow-up duration, inferences cannot be readily made from the statistical analyses performed. Therefore, any observed associations should be interpreted cautiously, as they may be due to reporting bias, confounding, or random variation rather than true causal relationships. Third, the limited sample size restricts the generalizability of the study findings. Fourth, long-term follow-up was not uniformly reported across studies, limiting findings on recurrence, treatment durability, and long-term functional outcomes. Despite these limitations, this study presents a comprehensive review of the current literature on MAbC vertebral osteomyelitis to date. Furthermore, the presented case highlights the importance of a multidisciplinary management approach involving neurosurgery, infectious disease, radiology, and plastic surgery teams in achieving definitive diagnosis and tailored treatment of this rare and challenging multidrug-resistant infection. Additionally, by systematically reviewing the existing literature, this report contextualizes the present case within the broader spectrum of reported MAbC vertebral osteomyelitis and highlights recurring diagnostic and therapeutic challenges.

## 5 Conclusion

Overall, the available literature regarding MAbC vertebral osteomyelitis remains sparse and is composed primarily of isolated case reports and small case series with substantial heterogeneity in patient demographics, spinal levels involved, treatment strategies, and outcomes. Nevertheless, consistent themes emerge: delayed diagnosis due to non-specific presentation and negative routine cultures, frequent need for repeat tissue sampling, reliance on prolonged multidrug antimicrobial therapy, and a critical role for surgical intervention. This systematic review, together with the present case, underscores the importance of maintaining a high index of suspicion for MAbC in chronic or postoperative vertebral osteomyelitis and highlights the need for earlier recognition, standardized diagnostic pathways, and collaborative multidisciplinary care to optimize outcomes.

## Data Availability

The raw data supporting the conclusions of this article will be made available by the authors, without undue reservation.
