# Supplementary material for: Mycobacteroides abscessus complex vertebral osteomyelitis and discitis of the thoracolumbar spine: a systematic review of management and outcomes with illustrative case
Source: Front Cell Infect Microbiol. 2026 Jul 30;16:1867912. doi: 10.3389/fcimb.2026.1867912 (PMC13469649; doi:10.3389/fcimb.2026.1867912)
Supplement: Supplementary file 1 [file Table1.docx]

| **Table S1.** The search strategy of electronic databases | | |
| --- | --- | --- |
| **Database** | **Search Strategy** | **Number** |
| PubMed | #1: ("Spine"[Mesh] OR spine OR spinal OR vertebra*) AND ("Mycobacterium abscessus"[Mesh] OR mabc OR "mycobacterium abscessus") | 31 |
| Scopus | #1: ("Spine"[Mesh] OR spine OR spinal OR vertebra*) AND ("Mycobacterium abscessus"[Mesh] OR mabc OR "mycobacterium abscessus") | 57 |
| WOS | #1: ("Spine"[Mesh] OR spine OR spinal OR vertebra*) AND ("Mycobacterium abscessus"[Mesh] OR mabc OR "mycobacterium abscessus") | 34 |
|  | **Total:** | **122** |

3 December 2025

47 duplicates; 75 for screen
