# Supplementary material for: Mycobacteroides abscessus complex vertebral osteomyelitis and discitis of the thoracolumbar spine: a systematic review of management and outcomes with illustrative case
Source: Front Cell Infect Microbiol. 2026 Jul 30;16:1867912. doi: 10.3389/fcimb.2026.1867912 (PMC13469649; doi:10.3389/fcimb.2026.1867912)
Supplement: Supplementary file 2 [file Table2.docx]

Table S2. Risk of bias

| **Authors** | **Q1** | **Q2** | **Q3** | **Q4** | **Q5** | **Q6** | **Q7** | **Q8** | **Q9*** | **Q10*** | **Quality (Low, Moderate, High)** |
| --- | --- | --- | --- | --- | --- | --- | --- | --- | --- | --- | --- |
| Bashti et al., (2023) | Y | Y | Y | Y | Y | Y | Y | Y |  |  | High |
| Benjamin et al (2024) | Y | Y | Y | Y | Y | Y | Y | Y |  |  | High |
| Chan et al. (2001) | Y | N | Y | Y | Y | Y | Y | Y |  |  | High |
| Edwards et al. (2012) | Y | N | Y | Y | Y | NA | Y | Y |  |  | Moderate |
| Farajzadeh Sheikh et al. (2017) | Y | Y | Y | Y | N | N | N | N | Y | Y | Low |
| Farid et al., (2023) | Y | N | Y | Y | Y | Y | Y | Y |  |  | High |
| Garcia et al., (2013) | Y | N | Y | Y | Y | Y | Y | Y |  |  | High |
| Je et al. 2012 | Y | N | Y | Y | Y | Y | N | Y |  |  | Moderate |
| Kadota et al., (2019) | Y | N | Y | Y | Y | Y | Y | Y |  |  | High |
| Kato et al., (2014) | Y | N | Y | Y | Y | Y | Y | Y |  |  | High |
| Maxson et al., 1994 | Y | N | Y | Y | Y | Y | Y | Y |  |  | High |
| Moral et al., (2019) | Y | N | Y | Y | Y | Y | Y | Y |  |  | High |
| Omori et al., (2021) | Y | N | Y | Y | Y | Y | Y | Y |  |  | High |
| Patel et al., (2024) | Y | N | Y | Y | Y | Y | N | Y |  |  | Moderate |
| Rahi et al., (2021) | Y | N | Y | Y | Y | Y | Y | Y |  |  | High |
| Rhodes et al., (2025) | Y | N | Y | Y | Y | Y | Y | Y |  |  | High |
| Shahi et al., (2020) | Y | N | Y | Y | Y | N | N | Y |  |  | Low |
| Silva et al., (2015) | Y | N | Y | Y | Y | Y | Y | Y |  |  | High |
| Singh et al. 2020 | Y | N | Y | Y | Y | Y | N | NA |  |  | Low |
| Zhang et al., (2018) | Y | N | Y | Y | Y | Y | Y | Y |  |  | High |
| Serling-Boyd et al., (2020) | Y | N | Y | Y | Y | Y | Y | Y |  |  | High |
| Ma et al. (2021) | Y | Y | Y | Y | Y | Y | Y | N | Y | Y | High |
| Sarria et al. (1998) | Y | N | Y | Y | Y | Y | N | Y |  |  | Moderate |

*for case series
