# Supplementary material for: Mycobacteroides abscessus complex vertebral osteomyelitis and discitis of the thoracolumbar spine: a systematic review of management and outcomes with illustrative case
Source: Front Cell Infect Microbiol. 2026 Jul 30;16:1867912. doi: 10.3389/fcimb.2026.1867912 (PMC13469649; doi:10.3389/fcimb.2026.1867912)
Supplement: Supplementary file 3 [file Table3.docx]

**Table S3. Timeline of events**

| **Date** | **Event** |
| --- | --- |
| 15 months prior to presentation at our institution | Index L4-L5 microdiscectomy for radicular symptoms secondary to L4-L5 herniated disc. |
| 14 months prior to presentation at our institution | Seroma and surgical incision dehiscence. |
| 11 months prior to presention at our institution | MRI showing L4-L5 osteomyelitis and discitis and a small abscess near the surgical site. Treated with an 8-week course of IV vancomycin after a CT-guided aspiration positive for *Corynebacterium*. |
| Nine months prior to presention at our institution | Progressively worsening symptoms. Repeat MRI showing interval worsening of the abscess and osteomyelitis and discitis (now 6 cm from 2-3 cm previously). Surgical wound washout was performed with negative intraoperative cultures. Dehiscence with drainage was noted at the superior aspect of his incision. 8-week course of IV vancomycin, IV cefepime, and oral clindamycin was prescribed. |
| One month prior to presention at our institution | Recurrence of back pain with MRI at OSH reportedly noting a residual or recurrent disc space infection with progressive destruction of the endplates at L4-L5 and a small abscess with inflammation extending to the lower margin of L3. |
| Day 1 | Presented to our institution for a higher level of care with poor appetite, malaise, back pain, and chills at the time of admission. |
| Day 7 | Surgical lumbar wound washout debridement. |
| Day 8 | PICC line placed; 6-week course of daptomycin and cefepime started. Bacterial cultures positive for *Staphylococcus pseudointermedius*; fungal, AFB, and universal PCR results pending. |
| Day 27 | Development of new onset of copious purulent discharge from the prior surgical drain site. |
| Day 29 | AFB cultures positive for *Mycobacteroides abscessus*. Antibiotic regimen changed to 4 drug regimen: amikacin, azithromycin, imipenem, and eravacycline. |
| Day 32 | Surgical lumbar wound exploration and washout. |
| Day 37 | Macrolide susceptibility cultures returned positive for susceptibility to macrolides (clarithromycin) with a non-functional *erm(41)* gene; imipenem discontinued per infectious disease. |
| Day 59 | New onset of purulent drainage from his midline lumbar surgical incision. |
| Day 68 | Surgical placement of antibiotic beads and closure of back wound by plastic surgery. |
| Day 93 | Surgical debridement of back wound, placement of 2 antibiotic spacers, and layered back wound closure by plastic surgery. |
| Day 114 | PICC line removed and all antimicrobials changed to PO medications. |
| Day 121 | Debridement and wound closure with local muscle flap by plastic surgery; AFB cultures finalized as negative. |
| Day 150 | Latest neurosurgery follow-up. |
| Day 235 | Latest infectious disease follow-up. |
